# Factors associated with HIV seropositivity among HIV-exposed infants enrolled in the prevention of mother-to-child transmission program in Mara Region, Tanzania: a mixed-methods study

**DOI:** 10.11604/pamj.2026.54.22.52925

**Published:** 2026-05-29

**Authors:** Christina Mollel, Alice Lakati, Bernard Njau, Fransiscko Fundi, Charles Edward

**Affiliations:** 1 School of Public Health, Amref International University, Nairobi, Kenya,; 2 Kilimanjaro Christian Medical University, Moshi, Tanzania,; 3 Mangaka District Hospital, Mtwara, Tanzania,; 4 Amref Health Africa, Dar es Salaam, Tanzania

**Keywords:** HIV-exposed infant, mother-to-child transmission, Tanzania, viral load, mixed feeding

## Abstract

**Introduction:**

Mother-to-child transmission (MTCT) of HIV remains a persistent public health challenge in sub-Saharan Africa, including Tanzania. Despite the scale-up of Prevention of Mother-to-Child Transmission (PMTCT) programs, HIV seropositivity among HIV-exposed infants (HEIs) continues to occur, reflecting gaps in service delivery, maternal adherence, and health system performance. This study aimed to determine factors associated with HIV serostatus among HEIs enrolled in PMTCT in Mara Region, Tanzania.

**Methods:**

a retrospective cross-sectional study with mixed methods was employed. Quantitative data were extracted from medical records of 253 mother-baby pairs enrolled in PMTCT at four health facilities in Mara Region (January 2021-January 2025). Descriptive statistics and logistic regression were conducted using SPSS. Qualitative data were collected through in-depth interviews with 12 healthcare providers and 10 caregivers. Thematic analysis was performed to explore contextual barriers and facilitators influencing PMTCT outcomes.

**Results:**

overall, 21/253 (8.3%) HEIs were HIV-positive. Detectable maternal viral load (AOR=3.12, 95% CI: 1.18-8.25, p=0.022), mixed feeding (AOR=6.45, 95% CI: 1.82-22.84, p=0.004), and poor follow-up adherence (AOR=5.24, 95% CI: 1.12-24.45, p=0.035) were significant predictors. Qualitative findings revealed fear of stigma, mixed feeding due to work pressures, and weak tracking systems as primary barriers.

**tConclusion:**

the 8.3% transmission rate exceeds the UNAIDS target of 4%. Findings underscore that biological factors and behavioural adherence drive transmission. Interventions must prioritise maternal viral suppression, enforce exclusive breastfeeding, and enhance retention in the PMTCT cascade.

## Introduction

HIV/AIDS remains a major global public health challenge, particularly among women of reproductive age and children. In 2023, approximately 39 million individuals were living with HIV globally, of whom about 1.7 million were children younger than 15 years [1]. Transmission of HIV from mother to child during pregnancy, childbirth, or breastfeeding remains the primary route of HIV infection among children [2]. In the absence of intervention, the likelihood of vertical HIV transmission ranges between 15% and 45%; however, effective Prevention of Mother-to-Child Transmission (PMTCT) strategies can reduce this risk to below 5% [3,4]. Over the last decade, the worldwide expansion of PMTCT services has resulted in significant reductions in new pediatric HIV infections [5,6].

Mother-to-child transmission of HIV has been largely eliminated in many high-income settings due to universal antenatal HIV testing, sustained antiretroviral therapy for pregnant and breastfeeding women, safe delivery practices, and prompt infant prophylaxis [4]. Despite global advances, progress has remained uneven [7]. Sub-Saharan Africa, home to about 12% of the global population but nearly 70% of people living with HIV, still contributes the largest share of new pediatric HIV infections [8,9]. Persistent shortcomings in ART coverage, delayed early infant diagnosis, and attrition along the PMTCT care continuum continue to impede progress toward global elimination goals [9].

Within sub-Saharan Africa, several countries have expanded PMTCT coverage but continue to report HIV seropositivity among HIV-exposed infants (HEI) [7]. Studies from South Africa, Nigeria, Uganda and Kenya show MTCT or HIV seropositivity rates among HEI ranging between about 5% and 15%, often linked to suboptimal maternal ART adherence, delayed initiation of ART, mixed feeding practices, low birth weight, and health-system bottlenecks such as stock-outs and limited EID capacity [10-13]. These findings highlight that, even where PMTCT programs are in place, children remain at risk if critical steps in the cascade are missed or poorly implemented.

In line with World Health Organization guidance, Tanzania has adopted national PMTCT policies that provide lifelong ART (Option B+) to all pregnant and breastfeeding women living with HIV and ensure routine early infant diagnosis through DNA-PCR testing [11]. Nationally, HIV prevalence among adults is about 4.4-4.7%, with women bearing a higher burden than men, and HIV prevalence among pregnant women is estimated at around 6-7% [7,14]. PMTCT services have been scaled up to most public facilities, contributing to declines in MTCT. Nonetheless, available data indicate that HIV transmission rates among HEI in Tanzania still range between roughly 5% and 12%, and pediatric HIV remains an important contributor to new infections in the country [8,15].

Regional-level evidence further suggests that performance and outcomes vary within the country. For example, a recent study conducted in the Eastern Lake Zone and Southern Highlands reported an MTCT prevalence of 2.48% when data from five regions were combined [16]. However, this aggregated analysis did not provide region-specific estimates or explore contextual determinants at the regional level. As a result, the true magnitude of HIV seropositivity among HIV-exposed infants in Mara Region remains inadequately understood, alongside the maternal, infant, and health system factors that may be sustaining residual transmission.

Mara Region is a predominantly rural area along Lake Victoria, characterised by high fertility, socio-economic vulnerabilities and cultural practices such as early marriage, polygamy and reliance on traditional healers, all of which may influence maternal health-seeking behaviours and adherence to PMTCT services [8,9]. Despite the presence of more than 300 facilities offering PMTCT services, structural challenges, including long distances to facilities, shortages of trained staff, and intermittent ART and reagent supplies, may compromise the effectiveness of the PMTCT cascade and continuity of care for mother-baby pairs.

Given the global push towards elimination of MTCT and Tanzania's commitment to reducing new pediatric HIV infections, there is an urgent need for up-to-date, region-specific evidence on HEI outcomes and their determinants. Accordingly, this study sought to determine the prevalence of HIV seropositivity and to assess maternal, infant, and health system determinants of HIV serostatus among HIV-exposed infants enrolled in PMTCT services in Mara Region.

## Methods

**Study design and setting:** this study employed a retrospective and cross-sectional design with a mixed-methods approach. The quantitative component involved a retrospective review of routinely collected PMTCT medical records from January 2021 to January 2025. This timeframe was selected to capture recent data following the eMTCT strategic second plan implementation and healthcare provider training on updated PMTCT protocols in 2024. The retrospective design enabled assessment of infant outcomes without influencing routine service delivery. The qualitative component comprised in-depth interviews with purposively selected healthcare providers and caregivers to explore contextual factors influencing service uptake, treatment adherence, and infant outcomes. Four high-volume health facilities (>1000 active ART clients each) were purposively selected: Butiama District Hospital, Bunda District Hospital, Nyasho Health Centre, and Nyerere District Hospital. Mara Region is located in northern Tanzania. The Region borders Kenya and Lake Victoria, has a population of approximately 2.3 million, and is characterised by high fertility (total fertility rate 5.4), predominantly rural livelihoods (farming and fishing), and over 340 healthcare facilities implementing PMTCT programs. The region faces significant healthcare challenges, including infrastructure gaps, ART supply issues, and healthcare worker shortages.

**Population and study criteria:** the study population comprised mother-infant pairs accessing PMTCT services at the four selected public health facilities in Mara Region from January 2021 to January 2025, including those who received continuous follow-up through the PMTCT program and whose infants reached 18 months of age during the study period. Inclusion criteria were: (1) mother-infant pairs enrolled in PMTCT services at participating facilities between January 2021 and January 2025; (2) infants who reached 18 months of age with documented final HIV outcome per national PMTCT guidelines; (3) records containing minimum required variables (maternal ART status and dates, infant prophylaxis, EID/DNA-PCR test dates and results, final HIV outcome). Exclusion criteria were: (1) records missing core variables required to assign final HIV outcome; (2) infants lost to follow-up before confirmatory testing at 18 months; (3) records that were severely damaged or unreadable; (4) for the qualitative component: mothers/caregivers or healthcare workers who declined consent.

### Sample size and sampling procedures

***Quantitative component:*** a census approach was employed, including all eligible PMTCT records meeting inclusion criteria from the four selected facilities. This exhaustive review strategy eliminated selection bias and ensured findings reflected actual PMTCT outcomes. From an initial review of 296 records, 43 were excluded due to incomplete infant HIV status (n=13), missing maternal ART adherence (n=10), loss to follow-up (n=12), infant death (n=3), or abortion (n=5). The final sample comprised 253 records distributed as follows: Butiama District Hospital 76 (30.0%), Bunda District Hospital 53 (20.9%), Nyasho Health Centre 64 (25.2%), and Nyerere District Hospital 60 (23.7%).

***Qualitative component:*** purposive sampling was used to recruit 22 participants from the same four facilities. Participants included 12 healthcare providers (4 PMTCT nurses, 2 clinicians, 2 ART pharmacists, 3 mother mentors, and 1 Care and Treatment Centre (CTC) in-charge) with 3-15 years of experience, and 10 caregivers of HEIs (5 with HIV-negative infants, 3 with HIV-positive infants, and 2 with LTFU infants), aged 22-44 years. Fourteen participants were from rural areas, eight from urban areas. Recruitment was assisted by facility in-charges and PMTCT focal persons. Semi-structured interviews were conducted until thematic saturation was attained.

### Data collection

***Quantitative data collection:*** a structured extraction tool adapted from Torokaa *et al*. [16] and national PMTCT/EID registers was used to retrieve data from patient records, including maternal characteristics (ART adherence, viral load, timing of HIV diagnosis), infant characteristics (feeding practice, Nevirapine prophylaxis, comorbidities, immunization status, EID test dates and results), health system factors (distance to facility, follow-up adherence, tracking systems), and the primary outcome (infant final HIV status at 18 months). Four trained data collectors (two women, two men) with prior PMTCT experience underwent two-day training on tool usage and standardisation. One day was spent at each facility. Regular supervisory checks were conducted, and missing data were verified through cross-referencing with multiple records.

***Qualitative data collection:*** semi-structured interview guides were developed covering experiences with PMTCT services, barriers to retention and adherence, perceptions about testing and breastfeeding, health-system challenges, and improvement suggestions. Interviews were conducted in Kiswahili by the principal investigator and one experienced assistant, both fluent in Kiswahili and English. Each interview lasted approximately 30-45 minutes. All interviews were audio-recorded, transcribed verbatim in Kiswahili, and professionally translated into English. Back-translation was performed by an independent translator to ensure accuracy.

### Data analysis

***Quantitative data analysis:*** data were analysed using SPSS version 27. Descriptive statistics summarised sample characteristics: means and medians for continuous variables (infant age, maternal age), frequencies and percentages for categorical variables (infant sex, ART adherence, viral load suppression, feeding practices, EID completion). Bivariate analysis using chi-square tests examined associations between independent variables (maternal, infant, health system factors) and the dependent variable (infant HIV serostatus). Variables with p<0.2 in bivariate analysis were entered into multivariate logistic regression to calculate adjusted odds ratios (AOR) with 95% confidence intervals. The Wald statistic evaluated the significance of regression coefficients; the Hosmer-Lemeshow test assessed model goodness-of-fit. Statistical significance was set at p<0.05.

***Qualitative data analysis:*** thematic analysis was conducted using NVivo 12 software. Transcripts were systematically coded using a combination of deductive coding (based on predefined themes: maternal factors, infant factors, health system factors) and inductive coding (emerging themes from participant responses). Initial open coding generated preliminary categories. Codes were refined iteratively and grouped into broader themes. Member checking, audio recording, verbatim transcription, and triangulation of data from caregivers and healthcare providers enhanced credibility and trustworthiness.

**Ethical consideration:** ethical clearance was obtained from the Amref International University Ethical Review Board and the Lake Zone Institutional Review Board (LZIRB). Authorisation to access patient data was granted by the Regional Administrative Secretary (Ref No FA.190/227/01S/104). Written informed consent was obtained from all interview participants. Confidentiality and anonymity were strictly maintained. Participants were informed of their right to withdraw at any time without consequences to their care.

## Results

**Participant recruitment flow:** a total of 296 medical records of HIV-exposed infants (HEIs) enrolled in PMTCT services between January 2021 and January 2025 were initially reviewed across four health facilities in Mara Region. After excluding 43 records due to incomplete infant HIV status (n=13), missing maternal ART adherence (n=10), loss to follow-up before 18 months (n=12), infant death (n=3), and pregnancy termination (n=5), the final quantitative sample comprised 253 eligible mother-baby pairs (Figure 1). For the qualitative component, 22 participants (12 healthcare providers and 10 caregivers) were purposively recruited from the same facilities.

**Figure 1:**
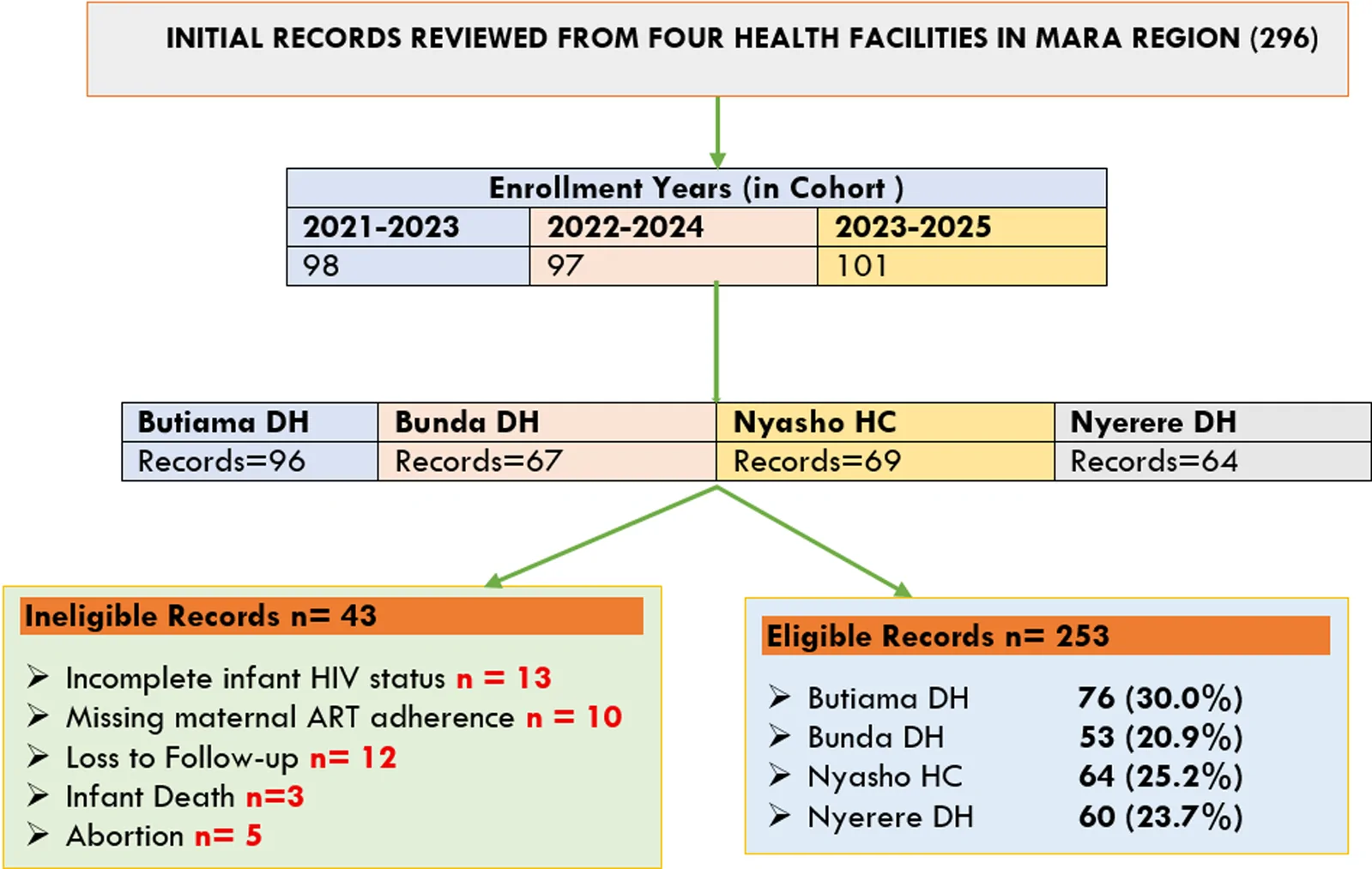
participant recruitment flow chart for quantitative component (N=296 initial, N=253 final)

**Demographic characteristics of the study population:** as shown in Table 1, among the 253 HEIs, 133 (52.6%) were female, and 120 (47.4%) were male, with a mean age of 3.2 years (SD±0.7). The majority of mothers (91.7%, n=232) were diagnosed with HIV before the current pregnancy, with good ART adherence documented in 84.2% (n=213) and viral load suppression (<50 copies/mL) achieved by 79.4% (n=201) during pregnancy. Regarding infant clinical characteristics, exclusive breastfeeding during the first six months was practised by 95.7% (n=242), and Nevirapine prophylaxis was received by 93.3% (n=236).

**Table 1:** characteristics of HIV-exposed infants and HIV testing cascade among mother-baby pairs enrolled in PMTCT program in Mara Region, Tanzania (N=253)

Variable	Category	Frequency (n)	Percentage (%)
Sex	Female	133	52.6
	Male	120	47.4
Age (Years)	Mean ± SD	3.2 ± 0.7	
1^st^ DBS (≤6 weeks)	Yes	221	87.4
	No	32	12.6
2^nd^ DBS (9 months)	Yes	193	76.3
	No	60	23.7
3^rd^ Rapid test	Yes	132	52.2
	No	121	47.8
Number of HIV tests	1	20	7.9
	2	46	18.2
	3	147	58.1
	4	40	15.8
Final HIV serostatus	Negative	232	91.7
	Positive	21	8.3
Maternal HIV diagnosis timing	Before pregnancy	232	91.7
	During pregnancy	17	6.7
	After delivery	4	1.6
Maternal ART adherence	Good	213	84.2
	Poor	40	15.8
Maternal viral load (pregnancy)	Suppressed (<50 copies/mL)	201	79.4
	Detectable (≥50 copies/mL)	52	20.6
Infant feeding practice	Exclusive breastfeeding	242	95.7
	Mixed feeding	11	4.3
Nevirapine prophylaxis	Yes	236	93.3
	No	17	6.7
NVP adherence	Good	232	91.7
	Poor	21	8.3
Place of delivery	Health facility	225	88.9
	Home	28	11.1
Immunization status	Complete	244	96.4
	Incomplete	9	3.6
Comorbidities	No	244	96.4
	Yes	9	3.6
Distance to facility	<5 km	105	41.5
	>5 km	148	58.5
PMTCT follow-up adherence	Good	242	95.7
	Poor	11	4.3
Missed appointment tracking	Yes	16	6.3
	No	237	93.7
Stigma/refusal documented	Yes	8	3.2
	No	245	96.8

**Prevalence of HIV seropositivity among HIV-exposed infants:** among the 253 HEIs included in the final analysis, 21 (8.3%) were HIV-positive at final confirmatory testing, while 232 (91.7%) were HIV-negative (Table 1). The testing cascade revealed declining engagement: first DBS uptake by six weeks was 87.4% (n=221), dropping to 76.3% (n=193) for the 9-month test, and only 52.2% (n=132) completed the final rapid test. A healthcare provider explained this decline. This 8.3% transmission rate exceeds the UNAIDS elimination target of less than 4%.

**Maternal and infant factors associated with HIV serostatus:** bivariate analysis (Table 2) revealed that poor maternal ART adherence (X^2^=5.12, p=0.024), detectable viral load (X^2^=7.89, p=0.005), and late HIV diagnosis (X^2^=4.56, p=0.033) were significantly associated with infant HIV positivity. Among infant factors, mixed feeding (X^2^=6.98, p=0.008), poor Nevirapine adherence (X^2^=5.67, p=0.017), home delivery (X^2^=4.89, p=0.027), incomplete immunization (X^2^=5.12, p=0.024), and presence of comorbidities (X^2^=3.98, p=0.046) were significantly associated. In the multivariate logistic regression model (Table 2), detectable maternal viral load (AOR=3.12, 95% CI: 1.18-8.25, p=0.022) and mixed feeding (AOR=6.45, 95% CI: 1.82-22.84, p=0.004) remained significant independent predictors. A mother of an HIV-positive infant explained the challenge of mixed feeding: *"I started giving porridge at two months because I had to return to work and had no one to help. The baby would cry, and I felt I was not producing enough milk."* This highlights how employment pressures, not lack of knowledge, drive mixed feeding practices.

**Table 2:** logistic regression analysis of factors associated with infant HIV serostatus among HIV-exposed infants enrolled in PMTCT program in Mara Region, Tanzania (N=253)

Variable	Category	HIV positive (n=21)	COR (95% CI)	p-value	AOR (95% CI)	p-value
**Maternal Factors**						
Timing of HIV Diagnosis	Before pregnancy (Ref)	16	1.00		1.00	
	During/after pregnancy	5	4.22 (1.40–12.71)	0.011	1.85 (0.52–6.58)	0.342
ART Adherence	Good (Ref)	14	1.00		1.00	
	Poor	7	3.01 (1.15–7.89)	0.025	1.52 (0.48–4.82)	0.478
Viral Load	Suppressed (Ref)	11	1.00		1.00	
	Detectable	10	3.82 (1.55–9.42)	0.004	3.12 (1.18–8.25)	**0.022**
**Infant Factors**						
Place of Delivery	Facility (Ref)	14	1.00		1.00	
	Home	7	5.02 (1.83–13.78)	0.002	2.12 (0.62–7.25)	0.232
Feeding Practice	EBF (Ref)	16	1.00		1.00	
	Mixed feeding	5	11.77 (3.65–37.9)	<0.001	6.45 (1.82–22.84)	**0.004**
NVP Adherence	Good (Ref)	15	1.00		1.00	
	Poor	6	5.79 (1.95–17.18)	0.002	2.84 (0.72–11.15)	0.134
Immunization	Complete (Ref)	17	1.00		1.00	
	Incomplete	4	10.68 (2.66–42.8)	0.001	3.15 (0.42–23.12)	0.265
Comorbidities	No (Ref)	18	1.00		1.00	
	Yes	3	6.28 (1.45–27.15)	0.003	1.95 (0.32–11.85)	0.468
**Health System Factors**						
Distance to Facility	<5 km (Ref)	5	1.00		1.00	
	>5 km	16	2.42 (0.86–6.81)	0.093	1.42 (0.38–5.32)	0.602
PMTCT Follow-up	Good (Ref)	16	1.00		1.00	
	Poor	5	11.77 (3.42–40.48)	<0.001	5.24 (1.12–24.45)	**0.035**
Stigma/Refusal	No (Ref)	19	1.00		1.00	
	Yes	2	3.96 (0.75–20.89)	0.105	2.98 (0.48–18.45)	0.241

COR: Crude Odds Ratio; AOR: Adjusted Odds Ratio; CI: Confidence Interval; EBF: Exclusive Breastfeeding; NVP: Nevirapine; Ref: Reference category. *Statistically significant p-values (<0.05) are shown in bold.

**Health system factors associated with HIV serostatus:** bivariate analysis (Table 2) showed that poor PMTCT follow-up adherence was significantly associated with infant HIV positivity (X^2^=6.23, p=0.013). Distance to health facility (>5 km) showed a higher proportion of HIV positivity (10.8% vs. 4.8%) but did not reach statistical significance (p=0.118). Documented stigma or refusal of care was present in only 3.2% (n=8) of records. Missed appointment tracking systems were documented in only 6.3% (n=16) of records. In the multivariate model (Table 2), poor PMTCT follow-up adherence remained a significant independent predictor (AOR=5.24, 95% CI: 1.12-24.45, p=0.035). A healthcare provider described the tracking gap: *"We know when mothers miss appointments, but we don't have a system to follow them up. There is no phone credit, no transport for home visits. We just wait for them to come back, and sometimes they never do."* This indicates that weak follow-up systems, not lack of awareness, drive poor retention.

### Perspectives of mothers, caregivers, and healthcare providers on factors influencing HIV serostatus

Four major themes emerged from thematic analysis.

***Theme 1: late antenatal care initiation:*** healthcare providers reported that many women presented to facilities only in the third trimester or after delivery, severely limiting the window for optimising ART and achieving viral load suppression before childbirth. A PMTCT nurse from Butiama District Hospital stated: *"Most of our mothers come late, some even after delivery. This delays access to ART and essential testing for both mother and baby."* A clinician from Nyasho Health Centre added, *"We have women who come for their first ANC visit at 32 weeks or later. By then, we have missed the critical window for early intervention."*

***Theme 2: ART adherence barriers:*** fear of disclosure, stigma, and lack of transport were frequently cited as reasons for missed doses. A mother mentor from Nyasho Health Centre observed: *"Some women stop taking ART when they feel better, or when they fear their partner will find out. They don't realise that stopping increases the risk for their baby." A caregiver shared: "There were days I didn't take my medication because I was afraid my sister-in-law would see me. I kept it a secret from everyone."* Healthcare providers noted that adherence challenges intensified after delivery, with mothers mistakenly believing the risk ends once the baby is born.

***Theme 3: mixed feeding practices:*** despite high rates of exclusive breastfeeding in quantitative data, qualitative interviews revealed that some mothers introduced other foods early due to employment pressures, lack of support, or perceived insufficiency of breast milk. A mother with an HIV-positive infant confessed, *"I started giving porridge at two months because I had to return to work and had no one to help. The baby would cry, and I felt I was not producing enough milk."* A PMTCT nurse from Nyerere District Hospital explained: *"When the baby has other illnesses, like pneumonia or diarrhoea, it weakens their immunity. If the mother missed doses or mixed feeds, the child is even more at risk."*

***Theme 4: Health system limitations:*** staff shortages, high workload, long distances to facilities, and weak tracking systems for mother-baby pairs emerged as significant barriers. A CTC in-charge from Butiama District Hospital stated: *"Yes, we offer services daily, but sometimes we have only one nurse covering everything-ANC, labor, and PMTCT. It's overwhelming. Mothers wait for hours, and some leave without being seen."* A caregiver from a remote village described: *"I have to walk for over an hour to reach the clinic. When it rains, I just stay home. There is no money for transport."* An ART pharmacist from Nyasho Health Centre noted: *"We know when mothers miss appointments, but we don't have a system to follow them up. There is no phone credit, no transport for home visits. We just wait for them to come back, and sometimes they never do."*

**Facilitators:** peer support groups, compassionate care, and community education were identified as key facilitators that could be leveraged to strengthen PMTCT services.

## Discussion

The present study found an HIV seropositivity prevalence of 8.3% among HIV-exposed infants enrolled in PMTCT services in Mara Region. This figure exceeds the UNAIDS elimination target of less than 4% [1]. In comparison, other Tanzanian studies reported lower rates: 5.4% in Dar es Salaam [17] and 4.8% in Northern Tanzania [18]. The higher rate observed in Mara suggests that rural regions face unique barriers to effective PMTCT implementation. These include longer distances to health facilities, limited healthcare infrastructure, and cultural norms that delay care-seeking. Similarly, a multi-regional study reported a combined MTCT prevalence of 2.48% across five Tanzanian regions, but that analysis did not provide region-specific estimates for Mara [16]. The substantial difference between this aggregate figure and our finding underscores the importance of localised data. When regions with stronger health systems are averaged with those facing greater challenges, the true burden in underserved areas becomes masked, potentially diverting resources away from where they are most needed. At the sub-Saharan Africa level, our findings align with studies from South Africa (8.2%), Nigeria (9.5%), and Uganda (6.5%), suggesting that while PMTCT programs have made significant progress, the elimination target remains elusive in resource-limited settings [11,19,20].

Turning to maternal factors, detectable viral load during pregnancy emerged as the strongest independent predictor of infant HIV positivity (AOR=3.12). This finding aligns with global evidence that viral suppression is the direct biological determinant of transmission risk [21]. When viral load is suppressed below detectable levels, transmission risk approaches zero; conversely, detectable viral load represents the point at which all other cascade failures manifest as risk. Poor ART adherence and late HIV diagnosis lost significance in the adjusted model, indicating that their effects operate through viral load. Qualitative findings further illuminate this pathway: fear of disclosure, stigma, and lack of transport were major barriers to consistent adherence. These observations are consistent with studies from Ethiopia and Tanzania [8,9]. Taken together, these findings suggest that adherence support must extend beyond medication counselling to address the social and structural determinants of adherence.

With respect to infant factors, mixed feeding during the first six months was the strongest infant-level predictor of HIV positivity (AOR=6.45). This finding is consistent with the landmark study by Coovadia *et al*., which demonstrated that mixed feeding increases postnatal transmission risk by disrupting gut integrity [22]. Qualitative findings revealed that work pressures and perceived milk insufficiency, not lack of knowledge, drove early introduction of complementary foods. Poor adherence to Nevirapine prophylaxis was also associated with HIV positivity. This is not surprising, as infant prophylaxis serves as a critical safety net when maternal ART adherence is compromised. Additionally, the presence of comorbidities in infants was significantly associated with HIV seropositivity, suggesting that weakened immune systems increase susceptibility to infection if exposure occurs [23,24].

When examining health system factors, poor PMTCT follow-up adherence remained a significant independent predictor (AOR=5.24). Retention is foundational to the cascade; without it, women cannot receive ART refills, infants cannot receive scheduled EID tests, and viral load monitoring cannot occur [25]. Qualitative narratives identified long distances, transport costs, and competing work commitments as barriers. Although distance to facility did not reach statistical significance in the quantitative analysis, the majority of participants lived more than 5 kilometres from a health facility, and qualitative findings confirmed it as a major obstacle. Staff shortages and weak tracking systems were also highlighted as significant constraints, with only 6.3% of records documenting any follow-up for missed appointments.

Finally, exploring the perspectives of mothers and healthcare providers revealed four key themes: late antenatal care initiation, ART adherence barriers (stigma, disclosure fear, transport), mixed feeding driven by work pressures, and health system limitations (distance, weak tracking). These qualitative insights explain the quantitative findings and reveal that barriers operate simultaneously at individual, family, health system, and community levels. Of particular concern, only 3.2% of medical records documented stigma or refusal of care, yet stigma was repeatedly cited as a pervasive barrier in interviews. This discrepancy indicates severe underreporting and suggests that health facilities must implement routine stigma screening and create safe spaces for disclosure counselling.

**Limitations:** the retrospective design limits control over confounding factors not documented in records. Social desirability bias may affect self-reported adherence. Findings may not be generalizable to facilities with different patient volumes or resource levels. However, the mixed-methods approach strengthens validity, and the census approach for quantitative data enhances representativeness.

## Conclusion

The 8.3% MTCT rate in Mara Region exceeds the UNAIDS elimination target of 4%. Detectable maternal viral load, mixed feeding, and poor follow-up adherence are independent predictors of HIV seropositivity among HEIs. Interventions must prioritise maternal viral suppression through enhanced adherence support, enforce exclusive breastfeeding through workplace accommodations and targeted counselling, and strengthen retention through active patient tracking systems. Addressing stigma and expanding peer support programs are essential to eliminating vertical transmission in Tanzania.

***What is known about this topic***

- *Effective PMTCT strategies can reduce MTCT risk to below 5%;*
- *Maternal viral load suppression is the primary determinant of vertical transmission;*
- *Mixed feeding before six months increases postnatal HIV transmission risk*.

***What this study adds***

- *Region-specific evidence from Mara Region showing 8.3% MTCT rate, exceeding the national average of 7.6%;*
- *Mixed feeding and poor follow-up adherence are independently associated with HIV positivity, even after adjusting for viral load;*
- *Qualitative insights reveal that work pressures and weak tracking systems, not lack of knowledge, drive these behaviours*.
